# Investigation of population structure in Gulf of Mexico *Seepiophila jonesi* (Polychaeta, Siboglinidae) using cross-amplified microsatellite loci

**DOI:** 10.7717/peerj.2366

**Published:** 2016-08-23

**Authors:** Chunya Huang, Stephen W. Schaeffer, Charles R. Fisher, Dominique A. Cowart

**Affiliations:** 1 Department of Biology, Pennsylvania State University, University Park, PA, United States; 2 Current affiliation: Department of Animal Biology, University of Illinois at Urbana-Champaign, Urbana, IL, United States

**Keywords:** deep sea, hydrocarbon seep, vestimentiferan tubeworm, siboglinid, microsatellite, population structure, gene flow

## Abstract

**Background:**

Vestimentiferan tubeworms are some of the most recognizable fauna found at deep-sea cold seeps, isolated environments where hydrocarbon rich fluids fuel biological communities. Several studies have investigated tubeworm population structure; however, much is still unknown about larval dispersal patterns at Gulf of Mexico (GoM) seeps. As such, researchers have applied microsatellite markers as a measure for documenting the transport of vestimentiferan individuals. In the present study, we investigate the utility of microsatellites to be cross-amplified within the escarpiid clade of seep vestimentiferans, by determining if loci originally developed for *Escarpia* spp. could be amplified in the GoM seep tubeworm, *Seepiophila jonesi*. Additionally, we determine if cross-amplified loci can reliably uncover the same signatures of high gene flow seen in a previous investigation of *S. jonesi*.

**Methods:**

Seventy-seven *S. jonesi* individuals were collected from eight seep sites across the upper Louisiana slope (<1,000 m) in the GoM. Forty-eight microsatellite loci that were originally developed for *Escarpia laminata* (18 loci) and *Escarpia southwardae* (30 loci) were tested to determine if they were homologous and polymorphic in *S. jonesi*. Loci found to be both polymorphic and of high quality were used to test for significant population structuring in *S. jonesi.*

**Results:**

Microsatellite pre-screening identified 13 (27%) of the *Escarpia* loci were homologous and polymorphic in *S. jonesi*, revealing that microsatellites can be amplified within the escarpiid clade of vestimentiferans. Our findings uncovered low levels of heterozygosity and a lack of genetic differentiation amongst *S. jonesi* from various sites and regions, in line with previous investigations that employed species-specific polymorphic loci on *S. jonesi* individuals retrieved from both the same and different seep sites. The lack of genetic structure identified from these populations supports the presence of significant gene flow via larval dispersal in mixed oceanic currents*.*

**Discussion:**

The ability to develop “universal” microsatellites reduces the costs associated with these analyses and allows researchers to track and investigate a wider array of taxa, which is particularly useful for organisms living at inaccessible locations such as the deep sea. Our study highlights that non-species specific microsatellites can be amplified across large evolutionary distances and still yield similar findings as species-specific loci. Further, these results show that* S. jonesi* collected from various localities in the GoM represents a single panmictic population, suggesting that dispersal of lecithotrophic larvae by deep sea currents is sufficient to homogenize populations. These data are consistent with the high levels of gene flow seen in *Escarpia* spp., which advocates that differences in microhabitats of seep localities lead to variation in biogeography of separate species.

## Introduction

Deep-sea hydrocarbon cold seep ecosystems are inhabited by macrofauna that directly or indirectly obtain nutrition from chemosynthetic processes in the absence of light (Sibuet & Olu, 1998; Bernardino et al., 2012). Cold seeps in the Gulf of Mexico were some of the first to be discovered (Kennicutt et al., 1988) and are home to the vestimentiferan tubeworms, sessile polychaete worms that lack digestive tracts as adults and rely upon internally located sulfide-oxidizing bacteria for nutrition (Childress & Fisher, 1992). The best-known vestimentiferan is the giant red tubeworm, *Riftia pachyptila* (Jones, 1981), which is endemic to hydrothermal vent ecosystems in the Pacific. Cold seep relatives of *R. pachyptila* belong to the genera *Seepiophila, Escarpia* and *Lamellibrachia*, which can be found at wide bathymetric ranges in the Gulf of Mexico and elsewhere (McMullin et al., 2010).

The species *Seepiophila jonesi* (Gardiner, McMullin & Fisher, 2001) commonly inhabits cold-seeps at depths between 500 m and 1,000 m along the upper Louisiana slope in the Gulf of Mexico. *Seepiophila jonesi* is a member of the monophyletic escarpiid clade of vestimentiferans, which are differentiated from the lamellibrachid clade based upon both morphological characters and phylogenetic investigations using the mitochondrial Cytochrome c Oxidase subunit I (COI) gene (Mcmullin et al., 2003; Andersen et al., 2004; Miglietta et al., 2010). A notable morphological character of *S. jonesi* is its prominent “growth rings”, flared funnel-like collars that are present along the length of its tube (see Gardiner, Mcmullin & Fisher, 2001 for photos). Also within the escarpiid clade is the genus *Escarpia*, including the species *Escarpia laminata* (Jones, 1985). *Escarpia laminata* is a deeper dwelling species found at depths ≥950 m across the Lower Louisiana Slope in the Gulf of Mexico and has been found in the same aggregations as *S. jonesi* at one site at 950 m (McMullin et al., 2003). *Escarpia laminata* lacks the growth rings seen in *S. jonesi*, instead having tubes with a smooth surface. *Seepiophila jonesi* also shares several characteristics with the lamellibrachid, *Lamellibrachia luymesi* (Van der Land & Nørrevang, 1975). *Seepiophila jonesi* and *L. luymesi* are frequently found in the same aggregations, house similar symbionts (Mcmullin et al., 2003), have identical motile larvae which are released into the water column (Hilário, Young & Tyler, 2005) and can live in excess of 200 years (Bergquist, Williams & Fisher, 2000).

Several studies have investigated tubeworm biogeographic ranges, population structure and connectivity, helping to clarify the mechanisms by which vestimentiferan larvae are transported to colonize disjointed deep sea habitats (Williams et al., 1993; Black et al., 1998; Van Dover et al., 2002; Hilário et al., 2011). Specific investigations focusing on the transport of larvae have used mathematic modeling, in conjunction with data on hydrodynamics and physiology of early life stages (see Marsh et al., 2001 for vents and Young, He & Emlet, 2012 for seeps). However, it is difficult to know when adults spawn, as well as where one must sample in the water column to document the transport of individuals. As such, researchers have additionally incorporated the use of genetic markers as a measure of dispersal of individuals (Black et al., 1998; Shank & Halanych, 2007; Coykendall et al., 2011).

Microsatellites, often referred to as short tandem repeats, have helped delimit populations and clarify dispersal patterns of seep vestimentiferans (McMullin et al., 2010; Cowart et al., 2013; Cowart et al., 2014). In addition to their prevalence in eukaryotic genomes, stretches of microsatellite repeats typically exhibit high mutation rates when compared to other markers, making them a frequent tool of choice in analyses of genetic structure in natural populations (Schlötterer & Pemberton, 1994; Ellegren, 2004). McMullin and co-workers (2010) amplified microsatellite loci in *S. jonesi* and *L. luymesi*, finding no evidence of isolation by distance among *S. jonesi*, despite detecting limited genetic isolation of one population of *L. luymesi*. To obtain these findings, McMullin used only a few polymorphic loci, all of which were species-specific to *S. jonesi* (seven loci) or *L. luymesi* (five loci). In addition to the expense of sampling at deep sea locations, the effort needed to develop primer sets for individual species can quickly become laborious and expensive, even with the aid of next generation sequencing technologies (Gardner et al., 2011). Accordingly, the ability to successfully develop “universal” microsatellite markers to amplify polymorphic and homologous products across species would reduce the costs associated to these analyses and allow researchers to investigate a wider array of taxa (Schlötterer, 2000).

Earlier work demonstrated that microsatellite markers can be successfully applied across species to clarify population boundaries in several vertebrate taxa, including birds (Primmer et al., 2005), frogs (Primmer & Merilä, 2002), land mammals (Moore et al., 1991), cetaceans (Schlötterer, Amos & Tautz, 1991; Valsecchi & Amos, 1996) and marine turtles (FitzSimmons et al., 1995). For microsatellite loci developed in cattle, the proportion of conserved loci decreased rapidly with increasing evolutionary divergence; 42% of polymorphic loci were amplified in sheep, but none were amplified in horse or human (Moore et al., 1991). Homologous polymorphic loci were also identified across eleven species of toothed and baleen whales, as well as in seven species within three families of marine and freshwater turtles, indicating a conservation of sequences over approximately 40 million years for cetaceans and 300 million years for the turtles (Schlotteröer, Amos & Tautz, 1991; FitzSimmons et al., 1995). The studies described above demonstrate that the transferability of microsatellite loci is uneven across taxonomic groups, but that variation is expected to yield high returns for taxa at close evolutionary distances and similar genome sizes (Barbará et al., 2007). More recently, microsatellites were developed and genotyped across several seep vestimentiferan species. Cowart et al. (2013) cross amplified nine polymorphic loci in three geographically disparate morphospecies of the genus *Escarpia*, supporting the occurrence of three genetically distinct groups, as well as panmixtic populations at the regional scale. Further, Cowart et al. (2014) amplified six polymorphic microsatellite loci across two species within the genus *Lamellibrachia* to clarify depth boundaries at Gulf of Mexico seeps.

In the present study, we investigate the utility of microsatellite markers originally designed for species of the genus of *Escarpia* to (1) be amplified in *Seepiophila jonesi*, which is also within the escarpiid clade of vestimentiferans and (2) determine if cross-amplified loci uncover the same signatures of low genetic structure seen in *S. jonesi* by McMullin et al., 2010. Our study includes not only additional polymorphic loci, but also more recently collected samples from additional locations in the Gulf of Mexico. If microsatellite loci amplified across greater evolutionary distances identify similar patterns of genetic structure, this suggests that these markers are “universal” and can be successfully implemented within the escarpiid clade to provide greater insights on the dispersal and biogeographic ranges of cold seep vestimentiferans.

## Methods

### Sample collection and preparation

**Figure 1:**
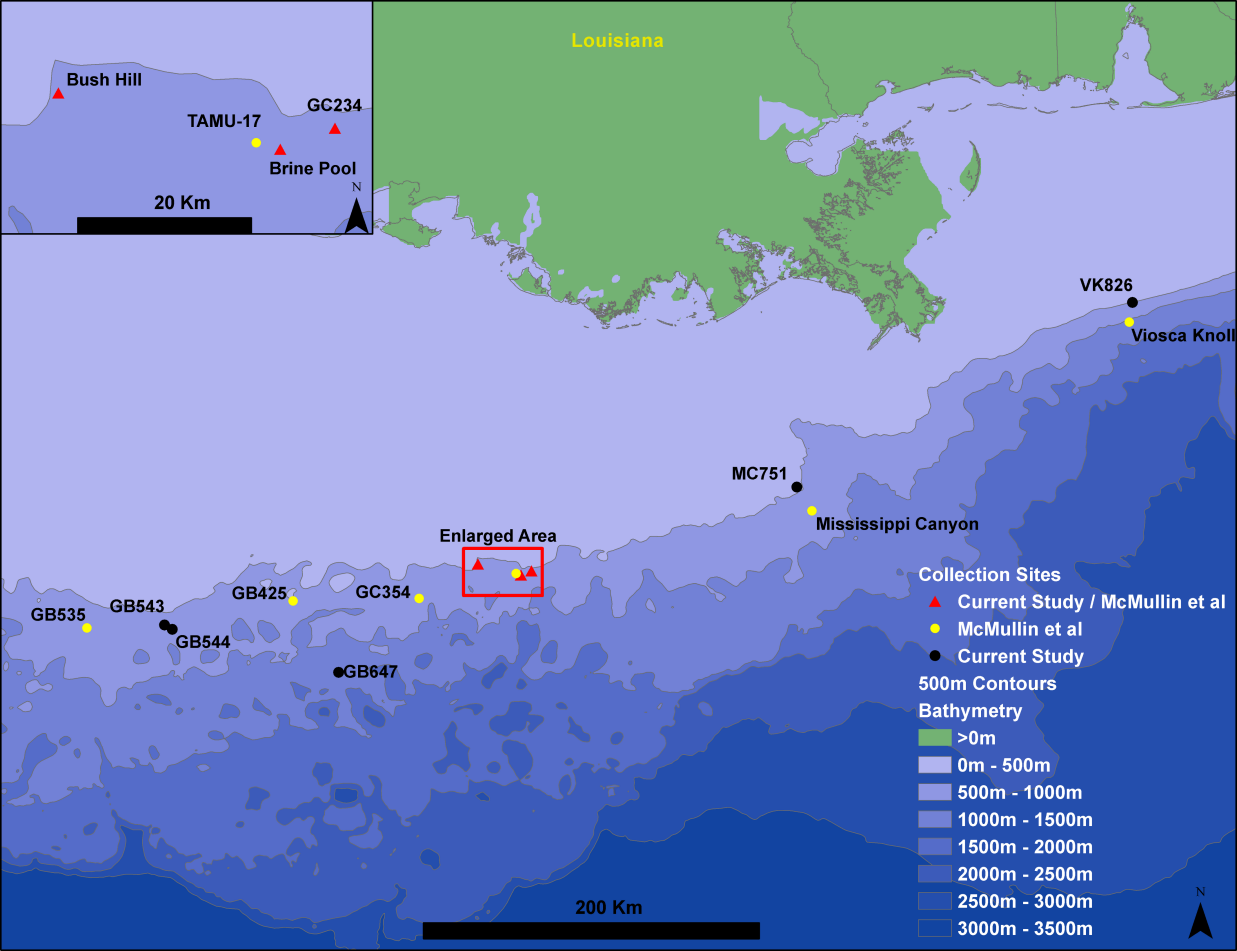
Gulf of Mexico seep locations from which *Seepiophila jonesi* individuals were collected. Seeps sites illustrated include those from the present study and McMullin et al. (2010). Sites are named for the Bureau of Ocean Energy Management Lease block in which they occur. The 500 m contours are from Geomapapp base + NOAA Coastal Relief Model—Gulf of Mexico (http://www.marine-geo.org/about/legal.php#credits, Divens, DL and Metzger, D).

*Seepiophila jonesi* individuals were collected from eight hydrocarbon seep locations across the Gulf of Mexico during several research cruises that occurred from 1995 to 2009 (Fig. 1 and Table 1). The map includes locations of *S. jonesi* collections that were made for our study, as well as McMullin et al. (2010). Collections were made with the aid of deep sea vehicles *Johnson Sea Link I*,* II* and *Alvin,* and remotely operated vehicle *Jason II*, operated aboard research vessels* Seward Johnson* (Harbor Branch Oceanographic Institution), *Atlantis* (Woods Hole Oceanographic Institution) and *Ronald Brown* (National Oceanic and Atmospheric Administration). Seep sites were all less than 1,000 m in depth and were between 5 and 541 km apart. Individual tubeworms were brought on board, morphologically identified as *S. jonesi*, dissected, and stored in −80 °C or 95% ethanol. Tissue samples were taken from the symbiont-free vestimentum tissue to avoid contamination from bacterial symbionts. The samples were transported to the laboratory at the Pennsylvania State University, where DNA extractions were performed according to the protocols described in Cowart, Huang & Schaeffer, 2012. No specific permissions were required to sample at any of these locations and the fieldwork performed here did not involve endangered or protected species.

**Table 1:** Locations of the eight seep sites sampled. Eight seep sites include the depth of collections, number of individuals collected (*N*) and regions. Regions are defined by Cairns et al. (1993) and McMullin et al. (2010).

Seep name	Latitude	Longitude	Depth (m)	*N*	Region
GB543	27.268°N	93.109°W	528	8	West
GB544	27.259°N	93.089°W	610	10	West
GB647	27.200°N	92.256°W	932	3	Central
Bush Hill	27.470°N	91.305°W	528–570	11	Central
Brine Pool	27.430°N	91.170°W	640	5	Central
GC234	27.447°N	91.134°W	527	12	Central
MC751	28.194°N	89.798°W	441	14	East
VK826	29.158°N	88.016°W	476	14	East

### Species confirmation

To confirm the identity of the putative *S. jonesi*, we amplified mitochondrial barcoding genes Cytochrome c Oxidase subunit 1 (COI) (Folmer et al., 1994) and large ribosomal submit rDNA (16S) (Kojima et al., 1995) for all 77 samples used in this study (Table S1, GenBank Accessions KT429444–KT429520). PCR reactions and gel electrophoresis were conducted as described in Miglietta et al. (2010). Purified PCR products were submitted to The Penn State Genomics Core Facility (University Park, PA) and run on a 3730XL DNA sequencer. Sequences were assembled and edited using Geneious Pro v5.5.5 (Biomatters Ltd.) and compared to the National Center for Biotechnology Information (NCBI) global nucleotide database via the Basic Local Alignment Search Tool (BLAST) blastn algorithm (Altschul et al., 1990). All sequences matched to *S. jonesi* at 99–100% identity to confirm the species. Pairwise identities of the alignments for each gene were >99%, with the percentage of identical sites >98%. Resulting haplotype networks identify a lack of separation by seep site, with the largest haplotypes for each gene being shared by individuals originating from several locations (Fig. S1). Together, these data support previous findings that there is low variation within mitochondrial genes of *S. jonesi* (Miglietta et al., 2010).

### Microsatellite development and analysis

Screening of microsatellite loci began by testing 48 loci that had shown cross-species amplification in *Escarpia laminata* (Gulf of Mexico) and *Escarpia southwardae* (Andersen et al., 2004, Gulf of Guinea). Previous testing performed by Cowart et al. (2013) indicated that these loci could have potential to successfully amplify in other members of the escarpiid clade of vestimentiferans, such as *S. jonesi*. The M13 tagging approach (Schuelke, 2000) was performed to test the 48 loci in two *S. jonesi* individuals taken from each of the eight locations, in order to determine if the loci were polymorphic. Loci were amplified using touchdown PCR conditions as described in Cowart, Huang & Schaeffer (2012), and fragment analyses were performed on the 3730XL DNA Sequencer using GeneScan with LIZ500 size standard (Applied Biosystems) at the Penn State Genomics Core Facility. Fragments were visualized using GeneMarker v.4.0 (Soft Genetics, State College, PA, USA) to remove loci that were monomorphic in *S. jonesi*.

Genotyped fragments were manually scored by eye, with the aid of GeneMarker. The Individual Inbreeding Model in INEST v.1.0 (Chybicki & Burczyk, 2009) and MICRO-CHECKER v2.2.3 (Van Oosterhout et al., 2004) were used to test for the presence of null alleles when a heterozygote deficiency was observed. Furthermore, large allele dropout and stuttering were tested to identify and correct genotyping errors in microsatellites that can cause deviations from Hardy–Weinberg proportions (Shinde et al., 2003). The false discovery rate (FDR) was computed with the statistical software R version 2.10.1; the *Q* value was used to adjust the probability cutoff for multiple tests of significant linkage disequilibrium and departures from Hardy–Weinberg Equilibrium (Storey, 2002; Storey & Tibshirani, 2003; R Development Core Team, 2009; Benjamini & Yekutieli, 2001). Finally, GENEPOP v4.1 (Raymond & Rousset, 1995) was implemented to test for genotypic linkage disequilibrium among loci. Microsatellite loci were removed either because null alleles had a frequency >20% or pairs of loci had strong non-random associations as measured by linkage disequilibrium (*P* < 0.05). As genotyping errors can lead to offsets by single base pair when determining allele size (Pompanon et al., 2005), we calibrated the automated allele calls produced from fragment analysis software with actual repeat lengths as described in Cowart et al., (2013). When the automated allele size was offset by less than one repeat, it was grouped with the nearest confirmed size.

We used Arlequin v3.5 (Schneider & Lischer, 2009) to test each microsatellite locus for departures from Hardy–Weinberg Equilibrium (HWE) and the presence of population structure by estimation of observed and expected heterozygosity within and between populations. The estimation of the probability that individuals from different sub- and total populations share the same alleles by descent (Wright’s *F*-Statistics) was calculated via FSTAT v2.9.3.2 (Wright, 1951; Goudet, 1995). Allelic and private allelic richness for each population was calculated using HP-RARE rarefaction method controlled for sample size (Kalinowski, 2005). STRUCTURE v2.3.x (Pritchard, Stephens & Donnelly, 2000; Pearse & Crandall, 2004) and EDENetwork v.2.16 (Kivelä, Arnaud-Haond & Saramaki, 2014) were implemented to visualize possible population structure among the sites within the Gulf of Mexico, by assigning individuals to populations and estimating allele frequencies. The STRUCTURE parameters were set as follows: assumptions of independent allele frequencies and admixed populations, three replicate simulations run with various *K* values, 100,000 Markov chain Monte Carlo (MCMC) repetitions for each cluster and burn-in of 10,000. EDENetworks assigns populations based on the distribution of genetic distances (links among populations and individuals) using the percolation theory (Stauffer & Aharony, 1994; Watts, 2004) without *a priori* hypothesis based on sampling locations. For analysis in EDENetworks, we used the shared allele distance (SAD), which resolves recent gene flow, to illustrate the genetic distances among individuals at a critical percolation threshold (Dpe, an inner property of the system) of 0.67 (Rozenfeld, Arnaud-Haond & Hernandez-Garcia, 2007; Rozenfeld et al., 2008). Finally, Analysis for Molecular Variance (AMOVA) (Excoffier, Smouse & Quattro, 1992) was calculated using GENODIVE v.2.0b22 (Meirmans & Hedrink, 2011).

## Results

### Microsatellite loci screening

Of the 48 loci tested in *Seepiophila jonesi*, 18 and 30 were originally developed for *Escarpia laminata* and *Escarpia southwardae*, respectively (Cowart, Huang & Schaeffer, 2012). The M13 screening process identified 18 of the 48 loci (37.5%) as polymorphic in *S. jonesi*. All other loci (62.5%) either did not amplify or were monomorphic and were discarded. The remaining loci (10 from *E. laminata* and eight from *E. southwardae*) were genotyped in all 77 *S. jonesi* individuals. Two additional *E. laminata* loci were discarded, as more than 30% of the individuals genotyped were unable to be scored due to low quality. Of the remaining 16 loci, three additional *E. laminata* loci were discarded due to high percentages of null alleles within *S. jonesi*, which can lower the observed heterozygosity values. None of the remaining 13 loci (five from *E. laminata* and eight from *E. southwardae*, Table S2) showed evidence of stuttering, large allele dropout, significant linkage disequilibrium or associations. Binned genotypes for these 13 loci were recorded in Tables S3–S15 and these loci were used for genetic diversity analyses.

### Allele frequencies and genetic diversity

Mean summary statistics across all 13 microsatellite loci amplified in 77 *S. jonesi* individuals from the eight sites are shown in Table 2. Across all sites, the mean numbers of alleles were between 3.3 at GB647 and 6.7 at VK826. In total, 131 alleles were detected across all loci. The smallest number of alleles (three alleles) occurred at locus ES454_22, while the largest number (24 alleles) occurred at locus ES454_31 (Table S16). The average number of alleles per locus for *S. jonesi* in this study was 10.1.

**Table 2:** Summary statistics for 13 microsatellite loci amplified in 77 individuals of *S. jonesi*.

Seep site	Summary statistics (means across all loci)							
	*N*_*A*_	*H*_*O*_	*H*_*E*_	*F*_IS_	AR	AR_SE_	*P*_AR_	${P}_{\mathrm{AR}}^{\mathrm{SE}}$
Bush Hill	5.769	0.635	0.657	0.035	3.2	0.339	0.25	0.089
GB647	3.385	0.590	0.703	0.193	3.38	0.368	0.3	0.159
GB544	5.462	0.618	0.627	0.016	3.14	0.344	0.21	0.072
GB543	5.231	0.599	0.657	0.094	3.29	0.370	0.27	0.101
GC234	5.538	0.635	0.657	0.045	3.15	0.316	0.31	0.113
Brine Pool	3.769	0.829	0.763	−0.102	3.21	0.421	0.22	0.083
VK826	6.692	0.635	0.677	0.064	3.35	0.350	0.38	0.094
MC751	6.231	0.712	0.710	−0.003	3.31	0.372	0.36	0.111

**Notes.**

Abbreviations: number of alleles observed across all loci (*N*_*A*_), observed (*H*_*O*_) and expected (*H*_*E*_) heterozygosity, Wright’s Inbreeding Coefficient (*F*_IS_), rarified allelic richness (AR) and standard error (AR_SE_), private allele richness (*P*_AR_) and standard error (${P}_{\mathrm{AR}}^{\mathrm{SE}}$). Rarified over six samples and means are not significantly different (*p* > 0.05).

Observed heterozygosity was lower than the expected heterozygosity across most loci, though these values were not significantly different from one another (Table S16). The mean observed heterozygosity frequencies were between 0.590 at GB647 and 0.829 at Brine Pool. Additionally, expected heterozygosity frequencies were similar across sites, with the values ranging from 0.627 at GB544 to 0.763 at Brine Pool. The low levels of heterozygosity seen here are similar to what was previously observed in *S. jonesi* (McMullin et al., 2010), other escarpiids (Cowart et al., 2013) and the lamellibrachids (Cowart et al., 2014), suggesting low levels of genetic variability as a result of inbreeding are common in seep vestimentiferans.

Allelic and private allelic richness, measures of genetic diversity, were found to be similar among seep sites. Allelic richness ranged between 3.14 at GB544 and 3.38 at GB647, while private allelic richness ranged from 0.21 at GB544 to 0.38 at VK826 (Table 2). Of the 104 population/locus combinations, there were 11 departures from HWE before corrections for multiple comparisons. However, after implementing the FDR correction, no individual combination departed significantly from HWE at the 0.05 level of significance.

### Genetic structure

To discern possible gene flow within and among seeps/populations, Wright’s inbreeding coefficient (*F*_IS_) was calculated (Table 2), along with the estimation of genetic differentiation across sites (*F*_ST_). Mean *F*_IS_ values ranged from −0.003 to 0.193 at MC751 and GB647, respectively (Table 2), which are values similar to those seen in *Escarpia* spp. tubeworms. Pairwise *F*_ST_ values across all loci are shown in Table S17. All *F*_ST_ values were below 0.03; the highest dissimilarity was seen between GB543 and VK826 (*F*_ST_ = 0.026), which are seep locations that are the farthest apart at 541 km. The *F*_ST_ values seen amongst *S. jonesi* are indicative of low levels of genetic differentiation (Hartl & Clark, 2007) and one cause of the homogenous population could be the presence of random mating amongst individuals.

STRUCTURE (*K* = 1) and EDENetworks analyses (Figs. S2 and S3) both identified a lack of genetic differentiation among *S. jonesi* collected from across the eight sites. AMOVA testing did detect significant structure among individual components (*P* = 0.001 Table 3), for which 28% of the total variance is explained. However, there was no significant structure detected among seep locations (*P* = 0.447 Table 3). The non-significant AMOVA results coupled with low *F*_ST_ values support that the eight seep locations are not significantly different from one another.

**Table 3:** Summary of Analysis of Molecular Variance (AMOVA) conducted for *Seepiophila jonesi* under the Stepwise Mutation Model (RST). Significance (∗) was tested using 1,000 permutations.

Source of variation	d. f.	SSD	Variation (%)	*P* value
Within individuals	77	94726.0	72.4	–
Among individuals	69	149832.6	27.6	0.001*
Among populations	7	15132.35	0	0.447

## Discussion

We sought to determine (1) whether microsatellite loci originally designed for species of the vestimentiferan genus *Escarpia* could be amplified in another escarpiid, *Seepiophila jonesi* and (2) if cross-amplified loci identified the same patterns of connectivity in *S. jonesi* that was previously documented by McMullin et al. (2010). Of the 18 and 30 loci originally developed for *E. laminata* and *E. southwardae*, 27.7% (five loci) and 26.6% (eight loci) were polymorphic and used for investigations of population structure in *S. jonesi*. Our findings demonstrate that microsatellites can be amplified across genera within the escarpiid clade to yield insights into the biogeographic ranges and evolutionary processes occurring in seep vestimentiferans.

### Cross-amplification and evolutionary distances

The success rate of cross-amplification has been found to be directly related to the evolutionary distance between taxa under consideration (Zane, Bargelloni & Patarnello, 2002; Primmer et al., 2005), with amplification success highest between species (within genus), followed by between genera (within family) and between families (within order) (see Barbará et al., 2007). Further, Primmer et al. (2005) employed the mitochondrial Cytochrome B (cytb) region as an indicator of cross-species transfer success, finding that analysis of pairwise distances was useful for predicting microsatellite amplification across a range of taxonomic groups.

The evolutionary history and relationships of vestimentiferans has been investigated extensively, primarily through the phylogenetic analysis of mitochondrial genes COI and 16S that identify slow rates of evolution within this group (Halanych, Lutz & Vrijenhoek, 1998; Black et al., 1997; McMullin et al., 2003; Miglietta et al., 2010; Vrijenhoek, 2013). Within the escarpiid clade, neither gene has been able to resolve three sister species of *Escarpia* (*E. laminata*, *E. southwardae* and *E. spicata*) as separate species, despite their differences in morphology and geographic distributions (Jones, 1985; Andersen et al., 2004; Miglietta et al., 2010). This evidence for incomplete lineage sorting prompted Cowart et al. (2013) to test the transferability of microsatellites originally developed for *E. laminata* to help clarify species boundaries for *Escarpia* spp. Seventy-two *E. laminata* loci were first tested in *E. southwardae*, with 30 (41.6%) successfully amplified and 12 (16.6%) as polymorphic. These 12 loci were then amplified in *E. spicata*, nine of which were polymorphic. This yielded nine loci that were successfully amplified and polymorphic in all three groups, allowing for inferences on species boundaries and connectivity of *Escarpia* spp. (Cowart et al., 2013). These findings suggest that both mtCOI and mt16S may be used as indicators of cross-taxa transfer success in seep tubeworms, much as ctyb has been used for bird taxa.

Earlier studies identified a conservation of microsatellite loci over vast evolutionary distances of approximately 40 million years for cetaceans and 300 million years for the turtles (Schlotteröer, Amos & Tautz, 1991; FitzSimmons et al., 1995). We presently lack estimates on the time of divergence between *Escarpia* and *Seepiophila*, however, conservative approximations have shown that vestimentiferans are a relatively young group that arose less than 100 million years ago (Black et al., 1997). Molecular clock analysis based on mtCOI suggests that vestimentiferans are an even younger group, arising less than 60 million years ago (Chevaldonné et al., 2002; Vrijenhoek, 2013). As the escarpiid clade falls within Vestimentifera, our findings support that microsatellites can be successfully transferred over evolutionary time of well under 60 million years. However, more nuanced estimates for escarpiid divergence and how far microsatellites may be transferred will require additional investigation.

While there are advantages to using microsatellites, repeat polymorphisms are subject to ascertainment bias, where loci developed for one taxonomic group may not be variable in related groups because they are shorter and possibly less polymorphic loci (Barbará et al., 2007; Hausdorf & Hennig, 2010; Vowles & Amos, 2006). Polymorphisms in microsatellites mainly occur as a result of variability in the length of sequence, rather than as mutations in the base pairs of the primary sequence (Ellegren, 2004). The most common polymorphisms are the gain or loss of a single repeat motif, as a result of DNA slippage events (Schlötterer & Pemberton, 1994; Schlötterer, 2000). If one were to apply a set of microsatellite loci to two different species, where only one species was used to develop the microsatellites, then one might obtain different estimates of variation in the second species. Therefore, one must check that levels of variation of microsatellite loci are the same between species before using them to estimate levels of gene flow, which is what we did with the *Escarpia* microsatellites tested in *S. jonesi*.

### Population genetics of Gulf of Mexico seep tubeworms

Our findings further support the presence of a single panmitic population of *S. jonesi* in the Gulf of Mexico, as previously identified by McMullin et al. (2010), a study based on fewer sites and seven species-specific microsatellites. In this respect, the cross-amplification of microsatellite loci can be a reliable and less costly alternative for providing insights into gene flow and population structure of seep vestimentiferans. The comparison of *S. jonesi* populations across seep sites and geographic regions identified *F*_ST_ values not significantly different from zero (Table S4), as well as a small variance of allele frequencies between collection sites, thus supporting that *S. jonesi* populations are genetically connected to others nearly 550 km away. These results are similar to the low levels of heterozygosity and the regional panmixia seen in *E. laminata* (Gulf of Mexico) and *E. southwardae* (Gulf of Guinea) (Cowart et al., 2013), indicating a high level of genetic exchange among seemingly isolated seep sites.

The panmixia amongst seep vestimentiferans is likely driven by multiple factors, including the stability of this environment, a moderately high larval dispersal potential and complex water currents that aid in larval dispersal. Cold seep ecosystems are more stable than the ephemeral and unpredictable hydrothermal vents; seeps thus encourage long term establishment of fauna by providing a relatively stable source of nutrients at numerous habitats across the Gulf of Mexico (Fisher et al., 1997; Sibuet & Olu, 1998; Bergquist et al., 2003). Additionally, several factors affect the dispersal ability and the population structure of seep species, including the seasonality of spawning, larval development, the settlement and longevity of reproductive adults (McMullin et al., 2010). Vestimentiferan tubeworms are sexually dimorphic and females store flame-shaped sperm bundles at the far posterior end of their reproductive tract (Hilário, Young & Tyler, 2005). Eggs are internally fertilized and are released into the water column, likely as a response to environmental cues (Vrijenhoek, 2010). Embryos develop into lecithotrophic larvae that utilize an energy source in the form of a yolk as they drift in the water column until suitable habitat is reached, whereupon they settle (Young et al., 1996). In the laboratory, *S. jonesi* larvae lived in the water column for 21 days before metamorphosis, indicating a possible dispersal distance of 40–60 km at depths less than 1,000 m in the Gulf of Mexico (Young et al., 1996; Tyler & Young, 1999).

Local water currents influence larval dispersal and are affected by a variety of circulation patterns and broad oceanic currents, including the Loop Current and its associated eddies (Oey, Ezer & Lee, 2005). Loop Current flows above 800 m in the Gulf of Mexico (Hamilton, 1990), which covers *S. jonesi* habitats to facilitate larval dispersal at these depths. Macdonald (1998) measured the velocities and directions of currents 200 m above the seafloor on the upper Louisiana slope and determined that tubeworm larvae could potentially disperse further than originally thought: up to 181 km during their approximate three-week larval lifespan. Young and co-workers (2012) ascertained that *Lamellibrachia luymesi,* which co-occurs in the same aggregations as *S. jonesi,* is capable of dispersing in both eastward and westward directions as far as 300 km in the Gulf of Mexico. This suggests that as *S. jonesi* larvae are exposed to similar currents, they may also reach distances of 300 m from their source populations. Interestingly, McMullin et al. (2010) identified differences in population structure between *S. jonesi* and *L. luymesi*, despite the many similarities between the two species (McMullin et al. 2003; Cordes et al. 2007; Hilário, Young & Tyler 2005). Differences in population structure could possibly be explained by the longevity of reproductive adults. *S. jonesi* and *L. luymesi* have been estimated to live in excess of 150 years (Bergquist, Williams & Fisher, 2000; Cordes et al., 2007); however, McMullin et al. (2010) observed that older-looking aggregations were only comprised of *S. jonesi*, suggesting that this species may have a longer reproductive lifespan than *L. luymesi*. Furthermore, differences between the two species may also result from dissimilarities in larval physiology or behavior. *L. luymesi* was found to be unable to tolerate the temperatures at depths above the thermocline, which narrows its biogeographic range in the Gulf of Mexico (Young, He & Emlet, 2012), though it is currently unknown if *S. jonesi* larvae have similar ranges of temperature tolerance. Larvae facing higher temperatures as they drift into shallow water may also be exposed to faster currents, which in turn, increase their metabolic rate and yolk depletion, while reducing dispersal time (Young, He & Emlet, 2012). The potential trade-off between metabolic rates and currents in shallow water may allow *S. jonesi* expand its dispersal range and habitat, but additional studies will be required to test this.

Overall, our study has demonstrated that not only can non-species specific microsatellite loci be amplified across larger evolutionary distances than initially realized, but these loci can provide valuable information on populations and dispersal of organisms living at diffuse deep sea habitats.

##  Supplemental Information

10.7717/peerj.2366/supp-1 Table S1 Metadata list of samples used in this study, including the collection site, sample year, dive number, collection vehicles, and Genbank Accession Numbers

10.7717/peerj.2366/supp-2 Figure S1 Median-joining haplotype networks of the COI (*n* = 30 sequences) and 16S (*n* = 47 sequences) genes Colors represent seep sites from which *Seepiophila jonesi* were collected. Sizes of haplotype circles are proportional to the number of individuals possessing the same sequence and each line represents one mutational change separating two haplotypes. Sequences were aligned using CLUSTALW (Thompson et al., 1994) implemented in MEGA 7 (Tamura et al., 2011). Alignments were imported into DNAsp v.5.10.1 (Rozas-Rozas et al., 1995) where identical sequences were clustered into haplotypes. Haplotype outputs were exported in Roehl format for network calculation and drawing in Network by Fluxus http://www.fluxus-engineering.com).

10.7717/peerj.2366/supp-3 Table S2 List of 13 microsatellite primers loci cross-amplified from *Escarpia* to *Seepiophila jonesi* All primers were originally developed and tested by Cowart, Huang & Schaeffer (2012).

10.7717/peerj.2366/supp-4 Tables S3–S15 Allele counts across eight sites per locus

10.7717/peerj.2366/supp-5 Table S16 Observed (top number) and expected (bottom number) heterozygosities for each locus at each seep

10.7717/peerj.2366/supp-6 Table S17 Pairwise F_ST_ values between sites for *S. jonesi*

10.7717/peerj.2366/supp-7 Figure S2 STRUCTURE results for *S. jonesi* in the Gulf of Mexico, based on 13 microsatellite markers Each vertical bar represents an individual tubeworm. The y-axis is the proportion of each individual’s genotype belonging to a distinct population cluster. In this case, every individual belongs to the same population cluster (*K* = 1).

10.7717/peerj.2366/supp-8 Figure S3 Network topologies of *S. jonesi* individuals with the shared allele distance based on 13 microsatellite markers Each node represents an individual tubeworm and nodes are labeled and color-coded by region. Only links with value smaller or equal to the percolation distance (*Dpe* = 0.67) are presented. All individuals are identified as one population cluster and various tested Dpe resulted in a similar clustering pattern.

10.7717/peerj.2366/supp-9 Supplemental Information 1 *S. jonesi* COI GenBank Submission

10.7717/peerj.2366/supp-10 Supplemental Information 2 *S. jonesi* 16S GenBank Submission

## Additional Information and Declarations

### Data Availability

The following information was supplied regarding data availability: National Center for Biotechnology Information (NCBI) GenBank Accessions KT429444–KT429520.
