# Supplementary material for: Investigation of population structure in Gulf of Mexico Seepiophila jonesi (Polychaeta, Siboglinidae) using cross-amplified microsatellite loci
Source: PeerJ. 2016 Aug 23;4:e2366. doi: 10.7717/peerj.2366 (PMC5012325; doi:10.7717/peerj.2366)
Supplement: Table S1 [file peerj-04-2366-s001.docx]

| **Sample Name** | **Collection Site** | **Sample Year** | **Dive Number** | **Collection Vehicle** | **I.D.** | **Gene** | **GenBank Accession #** |
| --- | --- | --- | --- | --- | --- | --- | --- |
| **S12-02** | Bush Hill | 1995 | 2615 | JSL | *S. jonesi* | COI | KT429445 |
| **S12-03** | Bush Hill | 1995 | 2865 | Alvin | *S. jonesi* | 16S | KT429474 |
| **S12-15** | Bush Hill | 1995 | 2857 | Alvin | *S. jonesi* | 16S | KT429485 |
| **S12-18** | Bush Hill | 1995 | 2865 | Alvin | *S. jonesi* | 16S | KT429488 |
| **S12-19** | Bush Hill | 1995 | 2865 | Alvin | *S. jonesi* | 16S | KT429492 |
| **S12-11** | Bush Hill | 1995 | 2853 | Alvin | *S. jonesi* | 16S | KT429481 |
| **S12-12** | Bush Hill | 1995 | 2853 | Alvin | *S. jonesi* | 16S | KT429482 |
| **S12-13** | Bush Hill | 1995 | 2853 | Alvin | *S. jonesi* | 16S | KT429483 |
| **S12-17** | Bush Hill | 1995 | 2853 | Alvin | *S. jonesi* | 16S | KT429487 |
| **S12-14** | Bush Hill | 1995 | 2853 | Alvin | *S. jonesi* | 16S | KT429484 |
| **S12-16** | Bush Hill | 1995 | 2857 | Alvin | *S. jonesi* | 16S | KT429486 |
| **S12-47** | Brine Pool | 1997 | 2850 | JSL II | *S. jonesi* | COI | KT429449 |
| **S12-51** | Brine Pool | 1997 | 2850 | JSL II | *S. jonesi* | COI | KT429450 |
| **S12-52** | Brine Pool | 1997 | 2850 | JSL II | *S. jonesi* | COI | KT429451 |
| **S12-53** | Brine Pool | 1997 | 2850 | JSL II | *S. jonesi* | COI | KT429452 |
| **S12-56** | Brine Pool | 1997 | 2850 | JSL II | *S. jonesi* | COI | KT429453 |
| **S12-07** | GB543 | 2003 | 4586 | JSL | *S. jonesi* | 16S | KT429477 |
| **S12-29** | GB543 | 2003 | 4586 | JSL | *S. jonesi* | 16S | KT429499 |
| **S12-30** | GB543 | 2003 | 4586 | JSL | *S. jonesi* | 16S | KT429500 |
| **S12-31** | GB543 | 2003 | 4586 | JSL | *S. jonesi* | COI | KT429447 |
| **S12-32** | GB543 | 2003 | 4586 | JSL | *S. jonesi* | 16S | KT429501 |
| **S12-33** | GB543 | 2003 | 4586 | JSL | *S. jonesi* | 16S | KT429502 |
| **S12-34** | GB543 | 2003 | 4586 | JSL | *S. jonesi* | 16S | KT429503 |
| **S12-35** | GB543 | 2003 | 4586 | JSL | *S. jonesi* | 16S | KT429504 |
| **S12-06** | GB544 | 2003 | 4585 | JSL | *S. jonesi* | 16S | KT429476 |
| **S12-20** | GB544 | 2003 | 4585 | JSL | *S. jonesi* | 16S | KT429490 |
| **S12-21** | GB544 | 2003 | 4585 | JSL | *S. jonesi* | 16S | KT429491 |
| **S12-22** | GB544 | 2003 | 4585 | JSL | *S. jonesi* | 16S | KT429492 |
| **S12-23** | GB544 | 2003 | 4585 | JSL | *S. jonesi* | 16S | KT429493 |
| **S12-24** | GB544 | 2003 | 4585 | JSL | *S. jonesi* | 16S | KT429494 |
| **S12-25** | GB544 | 2003 | 4585 | JSL | *S. jonesi* | 16S | KT429495 |
| **S12-26** | GB544 | 2003 | 4585 | JSL | *S. jonesi* | 16S | KT429496 |
| **S12-27** | GB544 | 2003 | 4585 | JSL | *S. jonesi* | 16S | KT429497 |
| **S12-28** | GB544 | 2003 | 4585 | JSL | *S. jonesi* | 16S | KT429497 |
| **S12-01** | GB647 | 2007 | 280 | Jason II | *S. jonesi* | COI | KT429444 |
| **S12-09** | GB647 | 2007 | 280 | Jason II | *S. jonesi* | 16S | KT429479 |
| **S12-10** | GB647 | 2007 | 280 | Jason II | *S. jonesi* | 16S | KT429480 |
| **S12-08** | GC234 | 2006 | 4587 | Alvin | *S. jonesi* | 16S | KT429478 |
| **S12-36** | GC234 | 2006 | 4587 | Alvin | *S. jonesi* | 16S | KT429505 |
| **S12-37** | GC234 | 2006 | 4587 | Alvin | *S. jonesi* | 16S | KT429506 |
| **S12-38** | GC234 | 2006 | 4587 | Alvin | *S. jonesi* | COI | KT429448 |
| **S12-39** | GC234 | 2006 | 4587 | Alvin | *S. jonesi* | 16S | KT429507 |
| **S12-40** | GC234 | 2006 | 4587 | Alvin | *S. jonesi* | 16S | KT429508 |
| **S12-41** | GC234 | 2006 | 4587 | Alvin | *S. jonesi* | 16S | KT429509 |
| **S12-42** | GC234 | 2006 | 4587 | Alvin | *S. jonesi* | 16S | KT429510 |
| **S12-43** | GC234 | 2006 | 4590 | JSL | *S. jonesi* | 16S | KT429511 |
| **S12-44** | GC234 | 2006 | 4590 | JSL | *S. jonesi* | 16S | KT429512 |
| **S12-45** | GC234 | 2006 | 4590 | JSL | *S. jonesi* | 16S | KT429513 |
| **S12-46** | GC234 | 2005 | 4859 | JSL | *S. jonesi* | 16S | KT429514 |
| **S12-05** | MC751 | 2009 | 3758 | JSL II | *S. jonesi* | COI | KT429446 |
| **S12-71** | MC751 | 2009 | 3758 | JSL II | *S. jonesi* | COI | KT429461 |
| **S12-72** | MC751 | 2009 | 3758 | JSL II | *S. jonesi* | COI | KT429462 |
| **S12-73** | MC751 | 2009 | 3758 | JSL II | *S. jonesi* | COI | KT429463 |
| **S12-74** | MC751 | 2009 | 3758 | JSL II | *S. jonesi* | COI | KT429464 |
| **S12-75** | MC751 | 2009 | 3758 | JSL II | *S. jonesi* | COI | KT429465 |
| **S12-76** | MC751 | 2009 | 3758 | JSL II | *S. jonesi* | COI | KT429466 |
| **S12-77** | MC751 | 2009 | 3758 | JSL II | *S. jonesi* | COI | KT429467 |
| **S12-79** | MC751 | 2009 | 3758 | JSL II | *S. jonesi* | COI | KT429468 |
| **S12-80** | MC751 | 2009 | 3758 | JSL II | *S. jonesi* | COI | KT429469 |
| **S12-81** | MC751 | 2009 | 3758 | JSL II | *S. jonesi* | COI | KT429470 |
| **S12-82** | MC751 | 2009 | 3758 | JSL II | *S. jonesi* | COI | KT429471 |
| **S12-83** | MC751 | 2009 | 3758 | JSL II | *S. jonesi* | COI | KT429472 |
| **S12-84** | MC751 | 2009 | 3758 | JSL II | *S. jonesi* | COI | KT429473 |
| **S12-04** | VK826 | 2009 | 3756 | JSL II | *S. jonesi* | 16S | KT429475 |
| **S12-57** | VK826 | 2009 | 3756 | JSL II | *S. jonesi* | COI | KT429454 |
| **S12-58** | VK826 | 2009 | 3756 | JSL II | *S. jonesi* | COI | KT429455 |
| **S12-59** | VK826 | 2009 | 3756 | JSL II | *S. jonesi* | COI | KT429456 |
| **S12-61** | VK826 | 2009 | 3756 | JSL II | *S. jonesi* | 16S | KT429515 |
| **S12-62** | VK826 | 2009 | 3756 | JSL II | *S. jonesi* | 16S | KT429516 |
| **S12-63** | VK826 | 2009 | 3756 | JSL II | *S. jonesi* | COI | KT429457 |
| **S12-64** | VK826 | 2009 | 3756 | JSL II | *S. jonesi* | 16S | KT429517 |
| **S12-65** | VK826 | 2009 | 3756 | JSL II | *S. jonesi* | COI | KT429458 |
| **S12-66** | VK826 | 2009 | 3756 | JSL II | *S. jonesi* | 16S | KT429518 |
| **S12-67** | VK826 | 2009 | 3756 | JSL II | *S. jonesi* | COI | KT429459 |
| **S12-68** | VK826 | 2009 | 3756 | JSL II | *S. jonesi* | COI | KT429460 |
| **S12-69** | VK826 | 2009 | 3756 | JSL II | *S. jonesi* | 16S | KT429519 |
| **S12-70** | VK826 | 2009 | 3756 | JSL II | *S. jonesi* | 16S | KT429520 |
