# Supplementary figures and images for: Investigation of population structure in Gulf of Mexico Seepiophila jonesi (Polychaeta, Siboglinidae) using cross-amplified microsatellite loci
Source: PeerJ. 2016 Aug 23;4:e2366. doi: 10.7717/peerj.2366 (PMC5012325; doi:10.7717/peerj.2366)

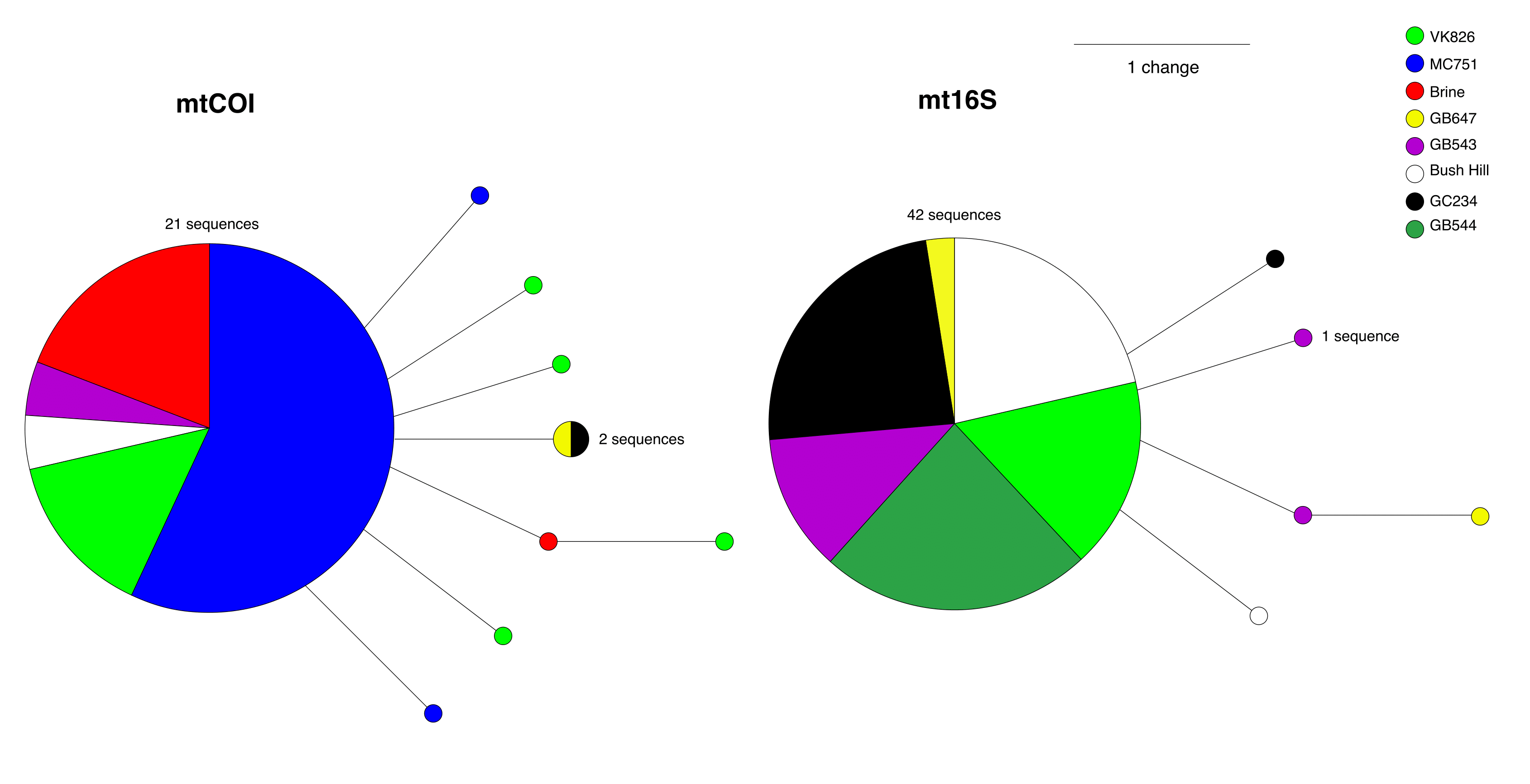

Supplement: Figure S1 — Colors represent seep sites from which Seepiophila jonesi were collected. Sizes of haplotype circles are proportional to the number of individuals possessing the same sequence and each line represents one mutational change separating two haplotypes. Sequences were aligned using CLUSTALW (Thompson et al., 1994) implemented in MEGA 7 (Tamura et al., 2011). Alignments were imported into DNAsp v.5.10.1 (Rozas-Rozas et al., 1995) where identical sequences were clustered into haplotypes. Haplotype outputs were exported in Roehl format for network calculation and drawing in Network by Fluxus http://www.fluxus-engineering.com). [file peerj-04-2366-s002.png]

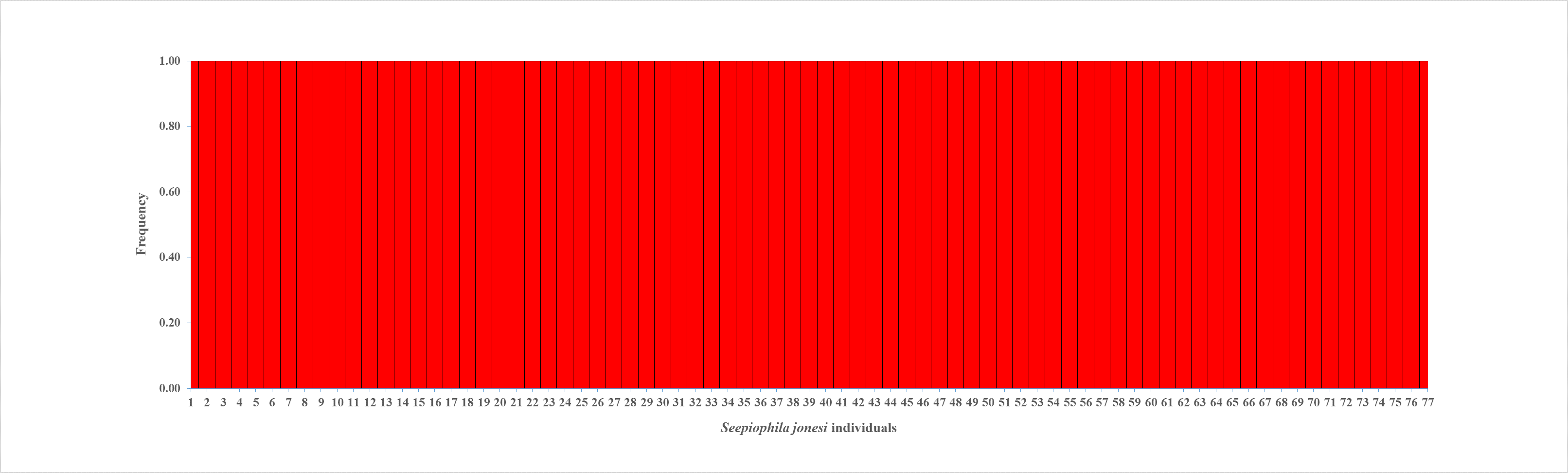

Supplement: Figure S2 — Each vertical bar represents an individual tubeworm. The y-axis is the proportion of each individual’s genotype belonging to a distinct population cluster. In this case, every individual belongs to the same population cluster (K = 1). [file peerj-04-2366-s007.png]

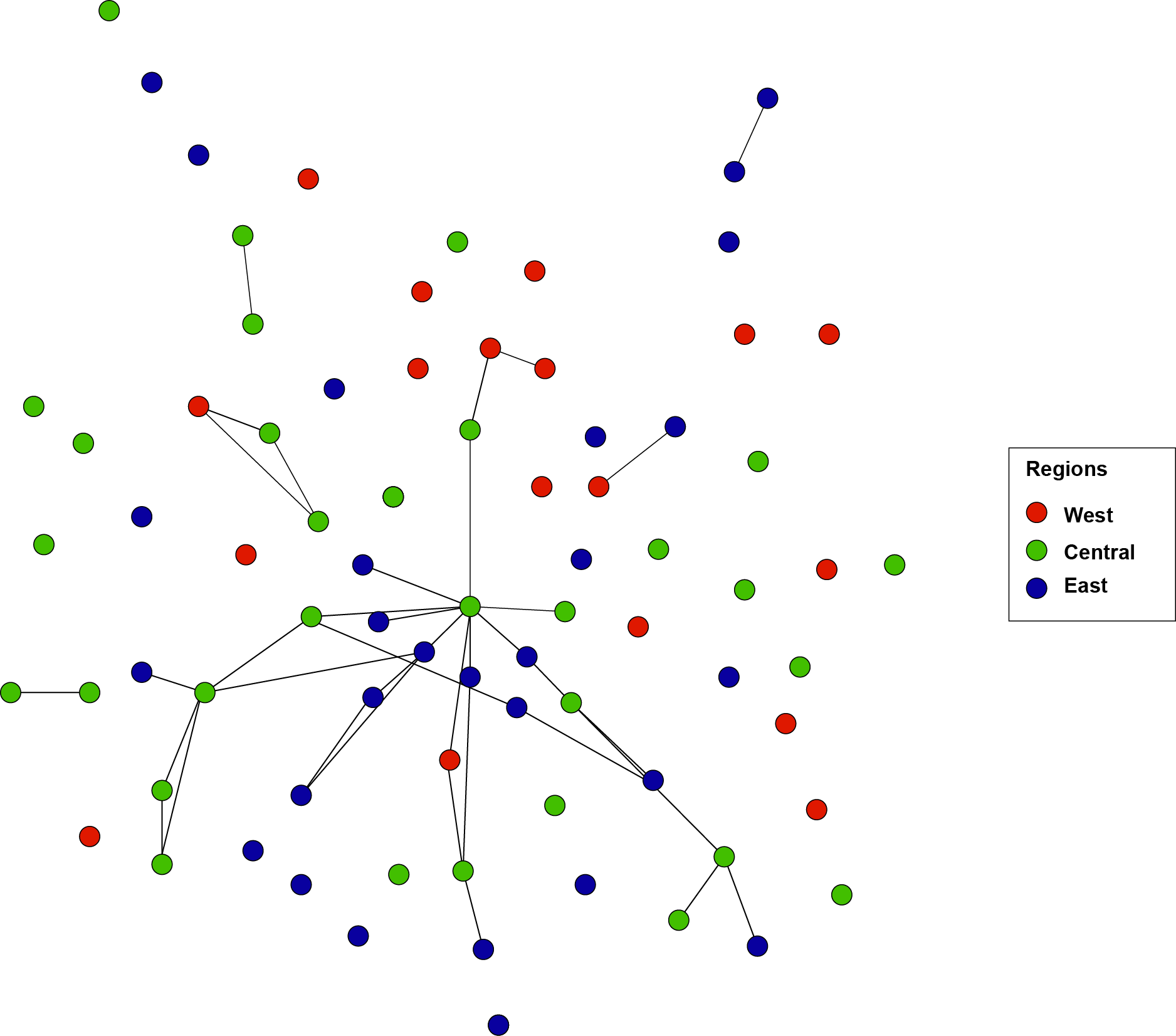

Supplement: Figure S3 — Each node represents an individual tubeworm and nodes are labeled and color-coded by region. Only links with value smaller or equal to the percolation distance (Dpe = 0.67) are presented. All individuals are identified as one population cluster and various tested Dpe resulted in a similar clustering pattern. [file peerj-04-2366-s008.png]
