# Supplementary material for: Investigation of population structure in Gulf of Mexico Seepiophila jonesi (Polychaeta, Siboglinidae) using cross-amplified microsatellite loci
Source: PeerJ. 2016 Aug 23;4:e2366. doi: 10.7717/peerj.2366 (PMC5012325; doi:10.7717/peerj.2366)
Supplement: Table S2 — All primers were originally developed and tested by Cowart, Huang & Schaeffer (2012). [file peerj-04-2366-s003.docx]

| **Locus** | **Primers (5’-3’)** | **Repeated motif** | **Pattern Size** | **Primer Product** **Size (bp)** | **Original Species** |
| --- | --- | --- | --- | --- | --- |
| EL454_2 | F: GAGACACCGTACGAATGACAGA  R: ACTGGAACTATGGCACGGAC | (TGTGCGCG)_16_ | 8 | 217 | *E. laminata* |
| EL454_5 | F: TGCATGTATACGTGGAGTGAGA  R: CCCTGCATTCATAATTGCTT | (TACATGCA)_18_ | 8 | 232 | *E. laminata* |
| EL454_6 | F: TGTGCTTCTCAAACCACC  R: CAAAAGGCTTTCTCGTCCAC | (CCACCAT)_20_ | 8 | 255 | *E. laminata* |
| EL454_54 | F: CGCCCTAAGCACTGTATTCC  R: TATAGACGCCAGGTGGAACG | (CACACG)_14_ | 6 | 173 | *E. laminata* |
| EL454_60 | F: CGCTCGTACGCACACTAATTT  R: TTGTGCTCCCATGGTGATAG | (GTTC)_26_ | 4 | 195 | *E. laminata* |
| ES454_4 | F: TTAACGATAACCGACCGACC  R: CTAAGCCTTTCGACTCGTGG | (TGTATGCG)_23_ | 8 | 142 | *E. southwardae* |
| ES454_13 | F: ATTCACCCACGCATTCTCTC  R: GAGTGGGTGAGTGCACGAG | (ACACACGC)_21_ | 8 | 178 | *E. southwardae* |
| ES454_18 | F: CACAAACGCGATGTAGGTGT  R: GTTGTTGCAGTGTGGACAGG | (GTGTGAGT)_20_ | 8 | 122 | *E. southwardae* |
| ES454_22 | F: ATCTAGCAGAAGTCAGGCCG  R: GCCTCAACCCTGACAGACAT | (TGCGGTG)_14_ | 7 | 106 | *E. southwardae* |
| ES454_31 | F: AATGAATTTCCCTCCCAAGG  R: TTCGATTGATGTCATGTCCG | (ACACGC)_28_ | 6 | 131 | *E. southwardae* |
| ES454_60 | F: TCCTCGTCAGACAATCCAAA  R: CCCAACCACGGGACACTAC | (TCCA)_34_ | 4 | 181 | *E. southwardae* |
| ES454_71 | F: AATGGGTACACAATACCGCC  R: AGGCAGATGAAACGGAGTGT | (TGTC)_25_ | 4 | 175 | *E. southwardae* |
| ES454_82 | F: AGTTCCCGACGGTGACATAC  R: GGTTCCGCGTAACAGCTAAA | (AAC)_18_ | 3 | 127 | *E. southwardae* |
