# Supplementary material for: Investigation of population structure in Gulf of Mexico Seepiophila jonesi (Polychaeta, Siboglinidae) using cross-amplified microsatellite loci
Source: PeerJ. 2016 Aug 23;4:e2366. doi: 10.7717/peerj.2366 (PMC5012325; doi:10.7717/peerj.2366)
Supplement: Table S16 [file peerj-04-2366-s005.docx]

|  | **Summary Statistics (means across all loci)** | | | | | | | | | | |
| --- | --- | --- | --- | --- | --- | --- | --- | --- | --- | --- | --- |
| **Loci** | **Allele #** | **Ho/** **H_E_** | **Mean** | **Bush Hill** | **GB647** | **GB544** | **GB543** | **GC234** | **Brine Pool** | **VK826** | **MC751** |
| EL454_2 | 15 | H_O_ | 0.763 | 0.800 | 0.667 | 0.800 | 0.750 | 0.583 | 0.800 | 0.846 | 0.857 |
|  |  | H_E_ | 0.777 | 0.847 | 0.800 | 0.779 | 0.825 | 0.649 | 0.778 | 0.766 | 0.767 |
| EL454_5 | 17 | H_O_ | 0.851 | 0.909 | 1.000 | 1.000 | 0.750 | 0.636 | 1.000 | 0.846 | 0.667* |
|  |  | H_E_ | 0.854 | 0.892 | 0.867 | 0.895 | 0.883 | 0.706 | 0.867 | 0.883 | 0.841 |
| EL454_6 | 5 | H_O_ | 0.534 | 0.545 | 0.333 | 0.400 | 0.500 | 0.636 | 0.500 | 0.571 | 0.786 |
|  |  | H_E_ | 0.692 | 0.675 | 0.733 | 0.700 | 0.575 | 0.693 | 0.750 | 0.675 | 0.653 |
| EL454_54 | 5 | H_O_ | 0.979 | 1.000 | 1.000 | 1.000 | 1.000 | 0.833 | 1.000 | 1.000* | 1.000 |
|  |  | H_E_ | 0.650 | 0.606 | 0.867 | 0.647 | 0.725 | 0.699 | 0.556 | 0.611 | 0.696 |
| EL454_60 | 4 | H_O_ | 0.630 | 0.545 | 0.333 | 0.500 | 0.875 | 0.636 | 1.000 | 0.615 | 0.538 |
|  |  | H_E_ | 0.554 | 0.515 | 0.333 | 0.595 | 0.625 | 0.502 | 0.679 | 0.591 | 0.618 |
| ES454_4 | 11 | H_O_ | 0.836 | 0.800 | 0.667 | 0.700* | 0.857 | 0.667* | 1.000 | 1.000 | 1.000 |
|  |  | H_E_ | 0.852 | 0.684 | 0.867 | 0.711 | 0.879 | 0.863 | 1.000 | 0.912 | 0.913 |
| ES454_13 | 12 | H_O_ | 0.782 | 0.818 | 0.667 | 0.800 | 0.750 | 0.909 | 0.600 | 0.857 | 0.857 |
|  |  | H_E_ | 0.806 | 0.836 | 0.533 | 0.784 | 0.858 | 0.887 | 0.756 | 0.902 | 0.839 |
| ES454_18 | 4 | H_O_ | 0.080 | 0.090 | 0.000 | 0.111 | 0.125 | 0.167 | --- | 0.071 | 0.071 |
|  |  | H_E_ | 0.167 | 0.255 | 0.533 | 0.111 | 0.125 | 0.159 | --- | 0.071 | 0.071 |
| ES454_22 | 3 | H_O_ | 0.071 | 0.090 | 0.000 | 0.000 | 0.000 | 0.250 | --- | 0.231 | --- |
|  |  | H_E_ | 0.293 | 0.455 | 0.533 | 0.209 | 0.264 | 0.424 | --- | 0.342 | --- |
| ES454_31 | 24 | H_O_ | 0.819 | 0.636* | 1.000 | 0.800 | 0.875 | 0.800 | 0.750 | 0.857 | 0.833 |
|  |  | H_E_ | 0.940 | 0.939 | 0.933 | 0.921 | 0.950 | 0.905 | 0.964 | 0.894 | 0.942 |
| ES454_60 | 6 | H_O_ | 0.614 | 0.700 | 0.667 | 0.800 | 0.375 | 0.500 | 0.800 | 0.429 | 0.643 |
|  |  | H_E_ | 0.612 | 0.537 | 0.800 | 0.679 | 0.425 | 0.493 | 0.711 | 0.672 | 0.603 |
| ES454_71 | 20 | H_O_ | 0.831 | 0.818 | 1.000 | 0.900 | 0.500* | 1.000 | 1.000 | 0.571* | 0.857 |
|  |  | H_E_ | 0.912 | 0.909 | 1.000 | 0.916 | 0.908 | 0.899 | 0.800 | 0.905 | 0.921 |
| ES454_82 | 5 | H_O_ | 0.398 | 0.500 | 0.333 | 0.222 | 0.429 | 0.250 | 0.667 | 0.357 | 0.429 |
|  |  | H_E_ | 0.446 | 0.395 | 0.333 | 0.209 | 0.495 | 0.344 | 0.533 | 0.574 | 0.646 |

Significant difference between H_O_ and H_E_ P < 0.01

--- Locus was monomorphic; no test was done
