# Supplementary material for: Investigation of population structure in Gulf of Mexico Seepiophila jonesi (Polychaeta, Siboglinidae) using cross-amplified microsatellite loci
Source: PeerJ. 2016 Aug 23;4:e2366. doi: 10.7717/peerj.2366 (PMC5012325; doi:10.7717/peerj.2366)
Supplement: Table S17 [file peerj-04-2366-s006.docx]

|  | **Bush Hill** | **GB647** | **GB544** | **GB543** | **GC234** | **Brine Pool** | **VK826** |
| --- | --- | --- | --- | --- | --- | --- | --- |
| **Bush Hill** |  |  |  |  |  |  |  |
| **GB647** | -0.01805 |  |  |  |  |  |  |
| **GB544** | -0.01115 | -0.02969 |  |  |  |  |  |
| **GB543** | -0.01116 | -0.01527 | -0.00201 |  |  |  |  |
| **GC234** | 0.00401 | 0.00105 | -0.01017 | -0.00453 |  |  |  |
| **Brine Pool** | 0.00437 | -0.00102 | -0.04200 | 0.00357 | 0.00318 |  |  |
| **VK826** | 0.00272 | 0.00842 | -0.00963 | 0.02606 | 0.01187 | 0.00357 |  |
| **MC751** | 0.00727 | 0.00095 | -0.01007 | -0.00363 | 0.00149 | -0.01377 | 0.01115 |
